# Spatial coherence and the persistence of high diversity in spatially heterogeneous landscapes

**DOI:** 10.1002/ece3.9004

**Published:** 2022-06-09

**Authors:** Ankit Vikrant, Susanne Pettersson, Martin Nilsson Jacobi

**Affiliations:** ^1^ 11248 Department of Space, Earth and Environment Chalmers University of Technology Gothenburg Sweden

**Keywords:** high dispersal, metacommunity, population dynamics, spatial heterogeneity, species richness

## Abstract

Our planet hosts a variety of highly diverse ecosystems. The persistence of high diversity is generally attributed to factors such as the structure of interactions among species and the dispersal of species in metacommunities. Here, we show that large contiguous landscapes—that are characterized by high dispersal—facilitate high species richness due to the spatial heterogeneity in interspecies interactions. We base our analysis on metacommunities under high dispersal where species densities become equal across habitats (spatially coherent). We find that the spatially coherent metacommunity can be represented by an effective species interaction‐web that has a significantly lower complexity than the constituent habitats. Our framework also explains how spatial heterogeneity eliminates differences in the effective interaction‐web, providing a basis for deviations from the area‐heterogeneity tradeoff. These results highlight the often‐overlooked case of high dispersal where spatial coherence provides a novel mechanism for supporting high diversity in large heterogeneous landscapes.

## Graphical abstract

This article investigates species‐rich metacommunities that are characterized by high dispersal and heterogeneity. It shows that large contiguous landscapes that provide such settings could facilitate the persistence of high species richness.

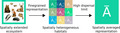

## 1 INTRODUCTION

Dispersal affects the population dynamics of species in many different ways. High connectivity between different habitats is known to synchronize population fluctuations, increasing the likelihood of global extinctions (Earn et al., 2000; Gokhale et al., 2018; Molofsky & Ferdy, 2005). If local dynamics allows for stable fixed points under high dispersal, then the persistence times of species increase with the number of patches (Yaari et al., 2012). Most studies have found intermediate dispersal to support highest levels of species richness, but these systems either comprised very few species (Gokhale et al., 2018; Molofsky & Ferdy, 2005) or did not consider complex interspecies interactions (Mouquet & Loreau, 2003).

Recently, the Generalized Lotka‐Volterra (GLV) equations have been widely used for the analysis of large ecological communities, providing novel insights into the generic properties of such communities using relatively few parameters (Barbier et al., 2018; Bunin, 2016). The use of GLV equations is often based on the random matrix approach pioneered by May (May, 1972) will who challenged the old view that diversity increases stability. May found that increasing complexity—defined in terms of the connectance and the variance of the random matrix of species interactions—results in less stable ecosystems (May, 1972). Recent works have studied dispersal between highly diverse communities using the GLV equations (Pearce et al., 2020; Roy et al., 2020), primarily focusing on, primarily focusing on endogenous population fluctuations for intermediate dispersal rates. Some other studies have reported autonomous species turnover using a spatially explicit model that described patch level dynamics through competitive Lotka‐Volterra equations. These studies also found many biodiversity patterns matching empirical data without invoking any structure in the underlying competitive interactions (O'Sullivan et al., 2019, 2021).

**FIGURE 1:**
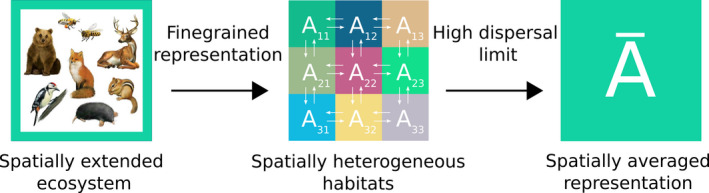
This schematic shows our modeling approach for large contiguous metacommunities. The spatially unresolved underlying system (left subfigure) is modeled as a set of connected heterogeneous patches that are characterized by local interaction matrices $A_{\left(\alpha \beta \right)}$. This metacommunity model admits a simplification in the high‐dispersal limit, i.e., the equilibrium densities can be estimated by an effective interaction matrix, which captures dynamics and properties of the metacommunity

A complementary formulation concerns high dispersal between habitats. High dispersal could lead to spatial coherence between habitats as in the population densities of species become constant everywhere. In the absence of dispersal, each habitat realizes the complexity of the interspecies interaction‐web (Pettersson et al., 2020). Large contiguous landscapes provide a relevant setting to study high‐dispersal scenarios since there could be considerable variation in habitats across space. This premise motivates the assumption that a sufficiently large landscape could be considered as a patchwork of many habitats connected through high dispersal (Figure 1). Previous studies investigating high‐dispersal rates in the context of the stability of communities indicate that high dispersal can shift May's complexity limit to higher complexities (Gravel et al., 2016); however, how dispersal affects actual population distribution and species richness remains unclear.

The extent of habitat heterogeneity is purported to have different possible relationships with the global species richness. The Area‐Heterogeneity Tradeoff (AHTO) states that given a fixed area, habitat heterogeneity has a positive influence on species richness except for very high heterogeneity levels that correspond to very small areas per habitat. However, a recent experimental test of this proposal reported a positive relationship even at the highest levels of heterogeneity (Ben‐Hur & Kadmon, 2020a). This result was attributed to the equalization of competitive ability between species when the deterministic effects of species interactions are stronger than stochastic extinctions (Ben‐Hur & Kadmon, 2020a). Heterogeneous landscapes have been reported to promote lower population variability partly due to dispersal between habitats (Oliver et al., 2010), which provides a greater scope for deterministic models of community assembly.

Motivated by the recent advances in the analysis of large complex communities (Barbier et al., 2018; Bunin, 2016; Pearce et al., 2020; Roy et al., 2020), we use the GLV equations with diffusion in discretized space to investigate the properties of metacommunities that become spatially coherent in the limit of high dispersal. The operational definition of a metacommunity that we use is closer to what was adopted by (O'Sullivan et al., 2019, 2021; Roy et al., 2020) in terms of having complex interspecies interactions. More generally, a metacommunity is defined as a collection of ecological communities that are linked by dispersal and may be heterogeneous in their biotic and abiotic attributes (Leibold & Chase, 2017). Metacommunities can be studied in various paradigms that differ in the amount of dispersal and the degree of environmental heterogeneity (Leibold & Chase, 2017; Leibold et al., 2004). Some of these paradigms do consider aspects of species traits, but the influence of complex interactions in local communities is generally not incorporated (Leibold & Chase, 2017).

For sufficiently high‐dispersal rates, we find that a spatially heterogeneous metacommunity can be considered as a well‐mixed system. Given local communities with many interacting species, we use this observation to demonstrate several heterogeneity scenarios that are analytically tractable. We discover a scenario that allows for fully feasible and stable communities even when the local complexity is arbitrarily high. The cause underlying this result also explains high diversity in more realistic heterogeneity settings that we further discuss. We also provide a theoretical exposition of deviations from the AHTO under high habitat heterogeneity and high dispersal between habitats.

## 2 MATERIAL AND METHODS

### 2.1 Generalized Lotka‐Volterra equations with diffusion

To investigate the effect of spatial heterogeneity in interactions on global species richness, we use the Generalized Lotka‐Volterra (GLV) equations with dispersal on a two‐dimensional grid:

(1) $$\begin{array}{c} \frac{\partial \phi_{i,\left(\alpha \beta \right)}}{\partial t}=r_{i}\phi_{i,\left(\alpha \beta \right)}\left(1‐\frac{\phi_{i,\left(\alpha \beta \right)}}{K_{i}}\right)+\phi_{i,\left(\alpha \beta \right)}\sum_{j=1}^{N}A_{ij,\left(\alpha \beta \right)}\phi_{j,\left(\alpha \beta \right)}\\ +D_{i}\left(\phi_{i,\left(\alpha +1\beta \right)}+\phi_{i,\left(\alpha ‐1\beta \right)}+\phi_{i,\left(\alpha \beta +1\right)}+\phi_{i,\left(\alpha \beta ‐1\right)}‐4\phi_{i,\left(\alpha \beta \right)}\right)/h^{2} \end{array}$$

where $\phi_{i,\left(\alpha \beta \right)}$ is the abundance density of species *i* at the spatial patch (α,β) in a two‐dimensional space. The discrete Laplace operator is the expression in parentheses multiplied by *D*, with the denominator *h* as the “distance” between habitats, which we set to 1. *r_i_* and *K_i_* are intrinsic growth rates and carrying capacities for species *i* respectively. The web of interspecies interactions for a pool of *N* species is represented by $A_{ij,\left(\alpha \beta \right)}$, which are *N* × *N* matrices with connectance (proportion of nonzero entries) *c* and their nonzero entries drawn from a normal distribution with variance $\sigma^{2}$ and mean μ. The diagonal entries of this matrix are set to zero. We vary the interaction strengths with spatial locations (αβ) although the mean and variance of the interaction strengths are kept constant. This variation characterizes the heterogeneity between habitats and is meant to capture differences in local abiotic conditions leading to spatially‐varying intensities in interactions between species.

We can represent different types of species communities by changing the mean of the species interaction strengths in $A_{ij,\left(\alpha \beta \right)}$. For a competitive community, for example, we use a sufficiently negative mean such that most interaction strengths are negative. A mean of zero (μ=0) is similarly used for a mixture of interactions. A mixture includes all types of interactions between pairs of species such as trophic (+,−), mutualistic (+,+), competitive (−,−), commensalistic (0,+), and amensalistic (0,−).

The standard deviation of interaction strengths, σ, can be used as a tuning parameter and proxy for complexity (if *N* and *c* are kept constant), as in May's classic paper (May, 1972). May's analysis used random community matrices *M* that were agnostic to equilibrium densities and any underlying dynamical equations. With reference to our model, the analog of *M* is the Jacobian at each patch of the homogenous system ($A_{ij,\left(\alpha \beta \right)}=A_{\mathrm{ij}}$) in the absence of dispersal. If the equilibrium densities of all species are positive, i.e., a feasible equilibrium, then the random interaction matrices *A* are related to the corresponding community matrices as *M* = ϕ*A, where ϕ* is a diagonal matrix with equilibrium densities on the diagonal.

For small σ in homogeneous systems ($A_{ij,\left(\alpha \beta \right)}=A_{\mathrm{ij}}$) without dispersal (*D* = 0), a system will reach a unique stable fixed point with complete coexistence (all *N* species nonextinct). If complexity (σ) is increased beyond a certain value, complete coexistence is lost and the system can no longer sustain all *N* species. To stay in a similar stable fixed point beyond this limit, single‐species extinctions would ensue. With continued increase of σ successively, more species go extinct. With further increase, the system is eventually left with no similar stable fixed points and it will transition to either oscillations, chaos, or a fixed point with a substantial loss of species (Pettersson et al., 2020). This latter transition is the transition that May referred to as the collapse and is the basis for his limit to the complexity an ecological community can sustain. The region between the first extinction and collapse is structurally unstable, meaning a small perturbation in parameters (*r_i_*, σ, *c,* etc.) leads to qualitative change, in effect species extinctions (Grilli et al., 2017; Pettersson et al., 2020; Rohr et al., 2014). This region can have multiple stable fixed points with differing species coexistence patterns (Kessler & Shnerb, 2015). The above described dynamical behavior of the GLV equations for a homogeneous system means that such a system with a species pool *N* and connectance *c* (and specified values of μ, $r_{i}$, and $K_{i}$) will have a certain species richness that depends on σ (the standard deviation of the entries of $A_{\mathrm{ij}}$).

Species are considered extinct if their density falls below 10^−5^ biomass units. We set $r_{i}=K_{i}=1$ for all species in all habitats. We also report our findings for the case when the carrying capacities and dispersal rates are allowed to vary across species (see Appendix S3).

### 2.2 High dispersal: Spatially extended versus well‐mixed systems

We study landscapes consisting of spatially contiguous habitats that allow high‐dispersal rates of species between them. High dispersal also motivates a new approach to look at large landscapes. Large contiguous landscapes are rarely homogeneous and could allow for sufficient variation in factors that affect species coexistence. Therefore, an ecosystem with a large spatial extent could be effectively understood as being comprised of heterogeneous patches with high dispersal between them.

In this high‐dispersal limit, the species abundance densities become coherent across spatial patches. The coherent equilibrium densities of a species differ very minutely across patches being equal up to a few decimal places. The differences become almost negligible if the dispersal rates are further increased. This happens despite the differences in interaction matrices that characterize these local communities. In this limit, we found that the GLV equations with dispersal (Equation 1) can be represented by regular GLV equations with an effective interaction matrix $\bar{A}$, as illustrated in Figure 1 (Also see Appendix S2 for proofs in one spatial dimension). In the coherent limit, the regular GLV equations describe the system as:

(2) $$\frac{d\phi_{i}\left(t\right)}{dt}=\phi_{i}\left(t\right)r_{i}\left(1‐\frac{\phi_{i}\left(t\right)}{K_{i}}\right)+\phi_{i}\left(t\right)\sum_{j=1}^{N}{\bar{A}}_{\mathrm{ij}}\phi_{j}\left(t\right),$$

where both the abundance densities $\phi_{i}$ and the effective interaction matrix ${\bar{A}}_{\mathrm{ij}}$ are independent of spatial location x. Each entry within this effective interaction matrix ${\bar{A}}_{\mathrm{ij}}$ is a spatial average over the corresponding entries in all the interaction matrices that represent local habitats.

However, interactions in the effective interaction matrix differ from the actual interactions between species at local patches, which are best captured by the local interaction matrices. This effective system captures the dynamics of the entire metacommunity in the high‐dispersal limit but is not a one‐to‐one representation of the local dynamics that eventually results in coherence. Although our results are based on dispersal rates that lead to spatial coherence, we found predictions of Equation 2 to hold even for lower dispersal that allows some spread in species abundances between local habitats.

The spatial heterogeneity and number of connected habitats determine the standard deviation of the interaction strengths in the effective interaction matrix. As explicitly shown in many studies using the GLV—and discussed above—the standard deviation of the interaction strengths is closely related to ecosystem stability and species richness. This means that the effective system can be analyzed—as in the framework of May—using the standard deviation of the entries of the effective interaction matrix ($\bar{\sigma }$) as the proxy for effective complexity. Thus, it follows that we can determine the stability and species richness of a heterogeneous spatial system in the coherent limit when we know $\bar{\sigma }$.

### 2.3 Effects of spatial heterogeneity on species richness

The relationship between spatial heterogeneity in interactions and the resulting species richness merits investigation in a variety of contexts. The simplest theoretical setting is that of extreme heterogeneity where each habitat corresponds to an independent random interaction matrix. This scenario is less likely in real systems, but it offers insights into the theoretical limits of species richness with regard to heterogeneity. Further, since interspecies interactions could vary in a very nonlinear way with abiotic factors such as temperature, (Steinbauer et al., 2018; Vandvik et al., 2020), these interactions might indeed have low correlation across habitats distributed along temperature gradients, for example. In addition to the independent random interaction matrices, we investigate a broad class of ecological settings, where habitats differ in interspecies interaction strengths but exhibit correlation in space. Varying the spatial correlation would result in different global species richness scenarios. We use a general set‐up that involves a different number of habitats in a landscape, such that these habitats are characterized by spatially correlated interaction matrices, and different spatial patches could have identical habitats. This set‐up is well‐suited to probe the relationship between habitat heterogeneity within a fixed area and global species richness when interspecies interactions have a greater influence over community assembly in comparison to environmental stochasticity.

More specifically, we study the effect that interaction heterogeneity has on the relationship between the number of habitats and species richness. We also discuss our findings in the light of the AHTO that posits a unimodal relationship (Allouche et al., 2012). Within AHTO, a decrease in species richness at high number of habitats is attributed to very small areas available per habitat, such that species would be more vulnerable to stochastic extinctions (Allouche et al., 2012; Bar‐Massada, 2015; Ben‐Hur & Kadmon, 2020b; de Souza Júnior et al., 2014; Kadmon & Allouche, 2007; Laanisto et al., 2013). However, a recent experimental test reported an increase in species richness even for very large number of habitats (Ben‐Hur & Kadmon, 2020a). Since that study expected dispersal to strengthen the positive relationship, high‐dispersal scenarios are conducive to comparisons against their work.

We use a spatial arrangement of nine patches, each of which could be represented by interaction matrices that characterize different habitats. The interaction strength corresponding to each species pair is correlated in space. For a given spatial correlation ρ, we plot the global species richness corresponding to increasing the number of habitats distributed randomly among the nine patches.

In some ecological settings, it is reasonable to expect higher correlations between adjacent spatial patches. We therefore also study this scenario by fixing correlations between adjacent spatial patches only. For simplicity, we restrict this analysis to one dimension to investigate how species richness of the entire system is affected by correlations that fall off with increasing distance. We also check how the variance of the effective interaction matrix depends on the number of spatial patches and the nearest‐neighbor correlation.

## 3 RESULTS

### 3.1 Heterogeneity‐richness relationships

**FIGURE 2:**
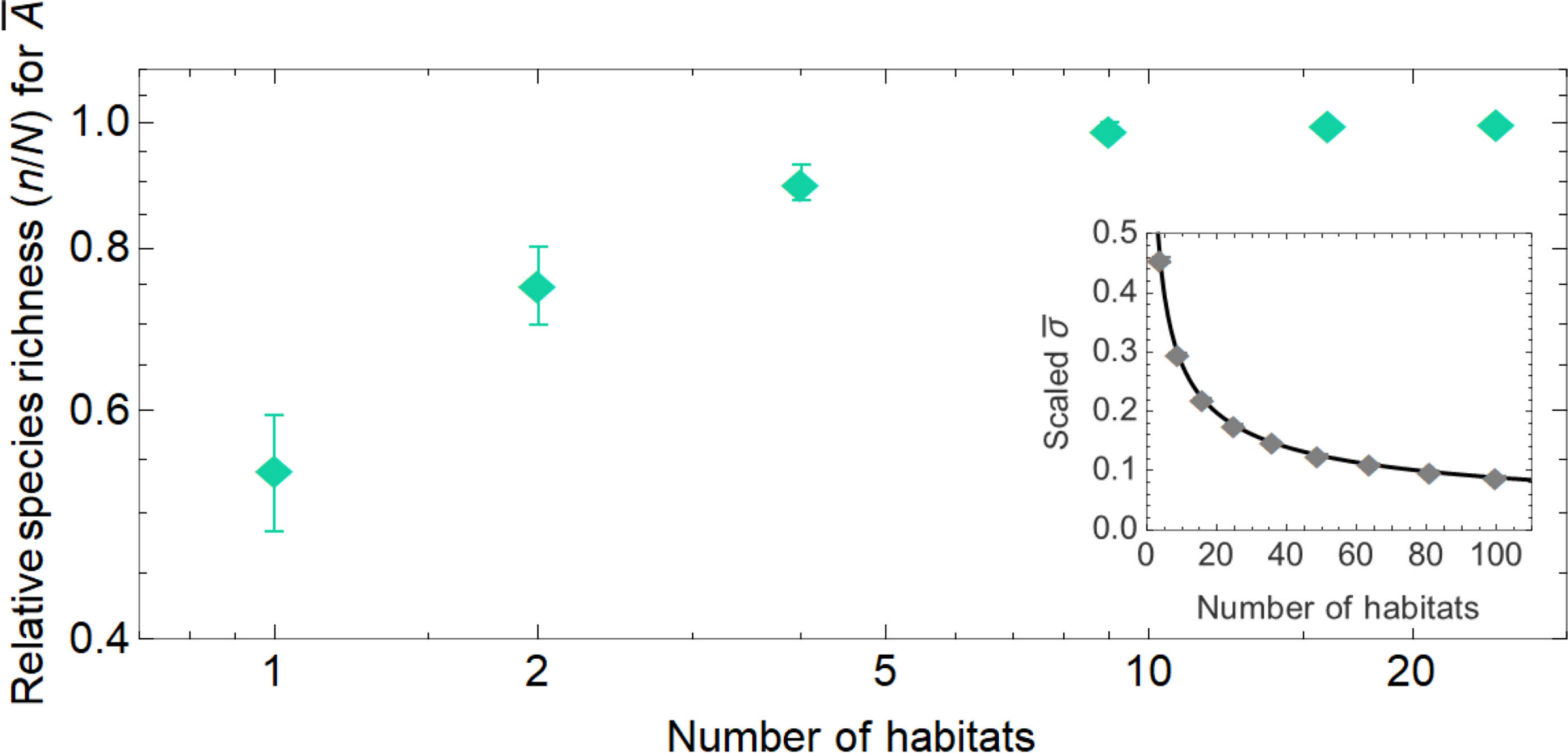
The plot shows how the relative species richness (*n*/*N*) increases with heterogeneity, in effect number of habitats. The data points are species richness averages from 20 runs of systems with $\mu_{A_{\left(\alpha \beta \right)}}=0$ and $\sigma_{A_{\left(\alpha \beta \right)}}=5/4\sqrt{\mathrm{cN}}$ and one standard deviation errorbars. The species richness saturates at relative species richness 1, which corresponds to complete coexistence of all species in the original species pool. The inset shows the decrease in the standard deviation of the entries of the effective interaction matrix $\bar{\sigma }$. Since the effective system captures the dynamics and stability properties of the underlying metacommunity, this demonstrates (as in the framework of May) how the proxy for effective complexity of the metacommunity decreases, thereby allowing for a higher species richness. We fix *c* = 1 and *N* = 50, although other values of *c* give similar results as long as the product of *c* and *N* is constant

In Figure 2 we show the case of high heterogeneity using unique random interaction matrices for each habitat. Figure 2 demonstrates the increase of species richness in the coherent stationary species density limit with increasing number of habitats. The species richness saturates at full coexistence, in effect at the number of species in the species pool *N*. The inset shows the decreasing standard deviation of the effective interaction matrix with increasing number of habitats. This decrease in standard deviation means a reduction in the effective complexity of the metacommunity while the actual local complexity is at a constantly higher level.

To find a theoretical expression for the variance ${\bar{\sigma }}^{2}$ and mean $\bar{\mu }$ of the effective interaction matrix, we view the nonzero entries in the habitat interaction matrices as stochastic variables $X_{\mathrm{ijg}}$, where the indices are *i* = *j* = 1,2,…*N* and *g* = 1,2…*G*, where *G* is the number of habitats. When the entries are drawn from a distribution with mean μ and standard deviation σ, the mean and standard deviation for the effective interaction matrix (which is the average of the local interaction matrices) can be found as

(3) $$\begin{array}{c} E\left[{\bar{A}}_{\mathrm{ij}}\right]=c\mu \\ =\bar{\mu }\\ Var\left[{\bar{A}}_{\mathrm{ij}}\right]=\frac{c}{G}\left(\sigma^{2}+\mu^{2}\left(1‐c\right)\right)\\ =\bar{\sigma }, \end{array}$$

where *c* is the connectance (see derivation in Appendix). The expressions in Equation 3 imply that in this extreme case of heterogeneity in the infinite habitat limit G→∞, the standard deviation of the average interaction matrix approaches zero $\bar{\sigma }\to 0$. In the context of stability limits, this result means that a metacommunity comprising an infinite number of habitats can support infinite complexity since the local σ of the habitats can be indefinitely large without any impending extinction or collapse. These results are in agreement with earlier studies that show dispersal to typically shift May's complexity limit (Gravel et al., 2016). Different network topologies for the spatial arrangement of habitats are consistent with these results as long as dispersal rates are high enough to guarantee coherence (see Section S5).

### 3.2 Spatial correlation mediates heterogeneity‐richness relationships

To obtain a theoretical expression for the variance of the metacommunity level effective interaction matrix when the interaction strengths across habitats are correlated, we follow the same procedure as for the uncorrelated case with one major difference. Since the interaction strengths across habitats are correlated, if an interaction is present (interaction strength nonzero) in some habitats, it will be present in all habitats. Thus, both the connectance and mean vanish from the expression. We then obtain the expression below

(4) $$\begin{array}{c} Var\left[{\bar{A}}_{ij\rho }\right]=\frac{\sigma^{2}}{G}\left(1+\left(G‐1\right)\rho \right)\\ ={\bar{\sigma }}_{\rho }^{2}. \end{array}$$

we see that the expressions in Equations 3 and 4 are equal for c=1 (all possible interactions present in all habitats) and ρ=0. The above expression for correlated habitats saturates to: $\rho \sigma^{2}$ in the limit of very large number of habitats *G*. This also implies an upper bound on the species richness for a given correlation between all habitats.

**FIGURE 3:**
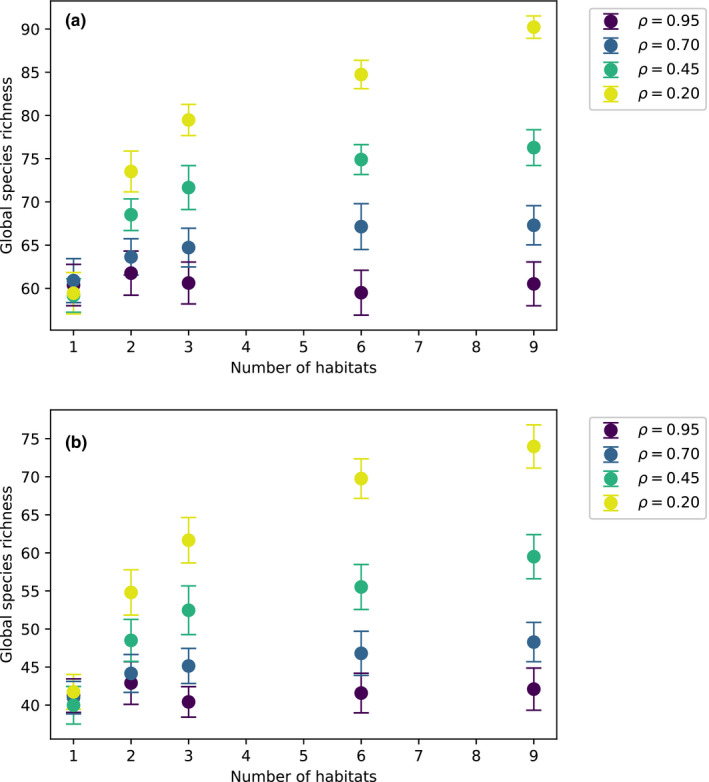
Relationship between habitat heterogeneity and species richness. For a given value of ρ, the plots show the mean species richness (with one standard deviation error bars) resulting from different number of habitats distributed over nine spatial patches. A given number of habitats correspond to an equivalent number of correlated random interaction matrices such that the means are computed over 50 realizations of these matrices. The two panels correspond to (a) mean interaction strength = 0, (b) mean interaction strength = −0.5. Both plots correspond to a pool of 100 species

Figure 3 shows the global species richness resulting from dynamics over different number of habitats. Although the interaction mean is zero in Figure 3 A as opposed to negative in Figure 3 B, both scenarios result in a significantly positive relationship between the global species richness and number of habitats for the lowest value of ρ. Although the stationary population densities are much lower in the negative mean case, high dispersal promotes many more coexisting species when interactions are highly heterogeneous across spatial patches. The significantly positive effect of a larger number of habitats in (Ben‐Hur & Kadmon, 2020a) could be explained by habitats differing greatly in the competitive interactions between species.

The nearest‐neighbor‐correlated habitat case introduces yet another theoretical expression for the variance of the effective interaction matrix, according to

(5) $$\begin{array}{c} Var\left[{\bar{A}}_{ij\rho_{\mathrm{nn}}}\right]=\frac{\sigma^{2}\left(G+2\sum_{\eta =1}^{G‐1}\rho^{\eta }\left(G‐\eta \right)\right)}{G^{2}}\\ ={\bar{\sigma }}_{\rho_{\mathrm{nn}}}^{2}. \end{array}$$

where $\rho_{\mathrm{nn}}$ is the nearest‐neighbor correlation. The correlation between neighbors falls off exponentially resulting in a correlation of $\rho_{2nn}=\rho_{\mathrm{nn}}^{2}$ for the next nearest neighbors, $\rho_{3nn}=\rho_{\mathrm{nn}}^{3}$ for the third nearest neighbors, and so on. At a certain number of habitats, the variance saturates at a level below the $\rho_{\mathrm{nn}}$, with an effective dynamics that corresponds to the fully correlated case discussed previously with correlation ρ. In the infinite habitat limit G→∞, we get ${\bar{\sigma }}_{\rho_{\mathrm{nn}}}\to^{\infty }0$, since the majority of habitats are effectively uncorrelated. This means that metacommunities with highly correlated interactions in nearby habitats can still harbor large interaction heterogeneity on the whole, which facilitates a high species richness. Theoretically, an infinite species richness follows for a large enough system, since correlations fall off to zero at large distances.

## 4 DISCUSSION

The analysis of ecological communities as spatially extended versus well‐mixed systems differs in many ways. Our framework provides a unique perspective in terms of reconciling the two contrasting perspectives in the high‐dispersal limit. A simple intuitive result is that the stationary species densities over a heterogeneous landscape could be effectively obtained from a spatially averaged interaction matrix for high‐dispersal rates. This result offers insights into the processes that promote coexistence in large complex metacommunities. The subsequent discussion is entirely based on metacommunities with high‐dispersal rates, unless other conditions are explicitly specified.

The first result concerns a large number of local communities that are characterized by spatially uncorrelated interaction matrices. Dispersal and heterogeneity have been proposed to support highly complex yet stable ecosystems (Gravel et al., 2016), but the spatially coherent representation shows a theoretically exact limit where stability emerges even in the face of unbounded complexity in terms of species richness.

We also demonstrated the effects that habitat heterogeneity has on species richness for a range of spatial correlations between local communities. Our findings also explain the significantly positive relationship between the number of habitats and species richness, which has been reported recently. As posited in (Ben‐Hur & Kadmon, 2020a), such a relationship could occur in situations where the positive effect of reduced competitive differences overcomes the likelihood of stochastic extinction. In general, the deterministic component of species interactions is purported to prevail over environmental stochasticity when there is a high variation in interspecies competition (Kramer & Drake, 2014). We surmise that the highly positive effect of habitat heterogeneity on species richness results from high spatial variation in competitive interactions, in line with our results. Low spatial correlation between pairwise interactions—or equivalently high spatial variation in these interaction strengths—results in very low variance in the spatially averaged interaction matrix even for competitive communities. This implies that on the global scale, competitive differences between species almost vanish within this limit. In fact, authors of Ben‐Hur & Kadmon, 2020a allude to the fact that fitness equalization between species should increase with increasing dispersal.

When correlations are assumed between the nearest‐neighbor habitats only, differences in interspecies interactions are lowered even for high correlation since the correlations fall off exponentially for habitats that are further apart. The positive effect on species richness also increases with the number of habitats consequently. We expect real metacommunities to be constrained by the number of habitats that could further be correlated differently depending on how they are spatially arranged. Even for such general settings, we still expect that higher habitat heterogeneity facilitates higher species richness.

Interspecies interactions are hard to measure in empirical studies, but it would help to explore their relationship with species traits and other biotic factors that serve as proxies for spatial variation in interactions. Such proxies have been used to explain the importance of interspecies interactions in understanding biodiversity responses along climate gradients (Vandvik et al., 2020). Abiotic factors such as temperature are known to be limited in explaining extinctions triggered by increased competition since interspecies interactions could have a highly nonlinear dependence on such factors (Steinbauer et al., 2018; Vandvik et al., 2020). With regards to environmental gradients, the likelihood of competitive exclusions is known to decrease from low to moderately stressful environmental conditions (Grime, 1973). Investigating how the interspecies interactions are spatially correlated along such gradients could better explain possible species richness scenarios, especially for high dispersal.

Dispersal over a large number of spatial patches is central to our analysis of highly diverse contiguous landscapes. Tropical lowland forests such as the Amazon provide good examples of such landscapes. There has been much debate around how such forests support high local (α) diversity of species such as trees (Valencia et al., 1994; Leigh Jr et al., 2004). The incidence of many generalist tree species in the Amazon is also unclear (Valencia et al., 2004). A recent phylogenetic study found that some diverse tree lineages are assembled by dispersal across Amazonia (Dexter et al., 2017). This study suggested that on evolutionary timescales, the entire Amazon basin should be considered as the metacommunity for local or regional tree communities. Therefore, widespread dispersal could precede speciation events in highly diverse landscapes lacking geographic barriers (Dexter et al., 2017).

High dispersal in large landscapes could be an overlooked precursor of high diversity that is further filtered by environmental conditions within the local communities. We analyzed a range of interaction heterogeneity scenarios to highlight the positive effect that heterogeneity has on species richness. Spatial heterogeneity could therefore be pivotal to facilitating highly diverse ecosystems in large contiguous landscapes.

## AUTHOR CONTRIBUTIONS

**Ankit Vikrant involved in** conceptualization (equal), formal analysis (equal), investigation (equal), methodology (equal), validation (equal), visualization (equal), writing—original draft (equal), and writing—review and editing (equal). **Susanne Marie Pettersson involved in** conceptualization (equal), formal analysis (equal), investigation (equal), methodology (equal), validation (equal), visualization (equal), writing—original draft (equal), and writing—review and editing (equal). **Martin Nilsson Jacobi involved in** conceptualization (equal), investigation (equal), methodology (equal), supervision (lead), validation (equal), and writing—review and editing (equal).

## CONFLICT OF INTEREST

The authors declare no conflict of interest.

## Supporting information

Appendix S1‐S3

## Data Availability

No new data were used in the preparation of this manuscript. Code availability: The associated code is available at https://github.com/ankitvi/Hetero‐coherence.
