# Supplementary material for: Spatial coherence and the persistence of high diversity in spatially heterogeneous landscapes
Source: Ecol Evol. 2022 Jun 9;12(6):e9004. doi: 10.1002/ece3.9004 (PMC9178367; doi:10.1002/ece3.9004)
Supplement: Supplementary file 1 — Appendix S1‐S3 [file ECE3-12-e9004-s001.pdf]

Online Supplement for:

Spatial coherence and the persistence of high diversity in spatially heterogeneous landscapes

Ankit Vikrant[1*†]

Susanne Pettersson[1†]

Martin Nilsson Jacobi[1]

1. Department of Space, Earth and Environment, Chalmers University of Technology, Maskingränd 2, 412 58 Gothenburg, Sweden;

∗ Corresponding author; e-mail: ankitv@chalmers.se.

† These authors contributed equally to this work.

S1 Extended Methods

In the limit of grid size going to zero $h \to 0$, the GLV equations in discretized space (Eq. 1 in main text) reduce to the continuous diffusion equations:

$$\begin{aligned} \frac{\partial \phi_i(\mathbf{x},t)}{\partial t} &= r_i \phi_i(\mathbf{x},t)\left(1 - \frac{\phi_i(\mathbf{x},t)}{K_i}\right) \\ &+ \phi_i(\mathbf{x},t)\sum_{j=1}^{N} A_{ij}(\mathbf{x})\phi_j(\mathbf{x},t) \\ &+ D\nabla^2\phi_i(\mathbf{x},t), \end{aligned} \tag{S1}$$

where $\phi_i(\mathbf{x},t)$, r_i and K_i are species abundance densities at position $\mathbf{x}$ and at time t, intrinsic growth rates and carrying capacities for species i respectively.

To numerically solve Eq. 1 in main text, we set D equal for all species and between all habitats at a rate high enough so that the local habitats are coherent this means in general $D \geq 10$. In most simulations we use $D \sim 1000$ to ensure coherence. The interaction matrices for each local habitat of the grid is $A_{ij,\alpha\beta}$. The discretized set of equations can then be solved using ODE solvers available in numerical computing suites. We implemented the procedure using *integrate.solve_ivp* in Scipy, and *DSolve* in Mathematica. The spatially coherent limit admitted almost exactly same stable fixed points at each patch, which we could verify in both the implementations. These stable fixed points are accurately approximated by the fixed point corresponding to the averaged interaction matrix (See section S2).

Periodic boundary settings were most often used in implementation, although in the high dispersal limit with coherent local habitats this gives the same results as reflective boundaries. Species are considered extinct if its density falls below 10^{-5} biomass units.

S2 Spatial coherence and the averaged interaction matrix

In the high dispersal limit, the effective dynamics is explained by the averaged interaction matrix. Here we sketch a simple proof of this in one spatial dimension. We begin by arguing that this result should hold even when the number of species and patches is low.

Consider two species over two patches. Species can only interact locally within each patch, but they can disperse between patches. The system of equations is:

$$\frac{\partial \phi_{i,\alpha}}{\partial t} = r_i \phi_{i,\alpha} \left(1 - \frac{\phi_{i,\alpha}}{K_i}\right) + \phi_{i,\alpha} \sum_{j \neq i} A_{ij,\alpha} \phi_{j,\alpha} + D\left(\phi_{i,\alpha+1} - \phi_{i,\alpha}\right) \tag{S2}$$

For simplicity, we set r and K equal to 1. The fixed point equations for species 1 are:

$$\phi_{1,1}\left(1 - \phi_{1,1}\right) + A_{12,1}\phi_{1,1}\phi_{2,1} + D\left(\phi_{1,2} - \phi_{1,1}\right) = 0 \tag{S3}$$

and

$$\phi_{1,2}\left(1 - \phi_{1,2}\right) + A_{12,2}\phi_{1,2}\phi_{2,2} + D\left(\phi_{1,1} - \phi_{1,2}\right) = 0 \tag{S4}$$

Now note that adding up the fixed point equations for one species always gets rid of the dispersal terms, irrespective of how many species or patches are present in the system. This implies that adding up the above equations for species 1 gives:

$$\phi_{1,1}\left(1 - \phi_{1,1}\right) + \phi_{1,2}\left(1 - \phi_{1,2}\right) + A_{12,1}\phi_{1,1}\phi_{2,1} + A_{12,2}\phi_{1,2}\phi_{2,2} = 0 \tag{S5}$$

Now consider equation S3. The reaction part of this equation, i.e., $\phi_{1,1}\left(1 - \phi_{1,1}\right) + A_{12,1}\phi_{1,1}\phi_{2,1}$ is usually of the order of 10^{-1} — 1 unless r and K take unreasonable values. This results in the simple observation that the magnitude of D determines how close $\phi_{1,2}$ and $\phi_{1,1}$ should be. A sufficiently large D drives the densities at the two patches very

close to one other, such that the densities are identical up to a few decimal places. The equations for species 2 result in the same inference. Then, using $\phi_{1,1} \sim \phi_{1,2}$ and $\phi_{2,1} \sim \phi_{2,2}$ in equation S5:

$$\phi_1 (1 - \phi_1) + \left(\frac{A_{12,1} + A_{12,2}}{2} \right) \phi_1 \phi_2 = 0 \tag{S6}$$

which is an effective fixed point equation for species 1 with an averaged interaction strength $\left(\frac{A_{12,1}+A_{12,2}}{2} \right)$. A similar equation holds for species 2. The same procedure can be extended for 2 patches containing any number of species. Note that the equilibrium densities are not exactly equal at every patch, but rather they are same up to many decimal places depending on how high the dispersal rate is.

It is possible to generalize this procedure to any number of patches in 1 spatial dimension. We write down the fixed point equations for species 1 in the following manner:

$$f_{1,\alpha} + D \left(\phi_{1,\alpha+1} + \phi_{1,\alpha-1} - 2\phi_{1,\alpha} \right) = 0 \tag{S7}$$

where $f_{1,\alpha}$ denotes the reaction term for species 1 at patch α. One can then write down the following equation for every pair of connected patches such that indices α and $\alpha - 1$ appear in the dispersal term:

$$2f_{1,\alpha} + f_{1,\alpha+1} - 2f_{1,\alpha-1} - f_{1,\alpha-2} + 5D \left(\phi_{1,\alpha-1} - \phi_{1,\alpha} \right) = 0 \tag{S8}$$

Equation S8 is obtained by multiplying fixed point equations corresponding to $f_{1,\alpha}$, $f_{1,\alpha+1}$, $f_{1,\alpha-1}$ and $f_{1,\alpha-2}$ with the relevant factors and summing them up. Note that adding up S7 for all patches results in

$$\sum_{\alpha} f_{1,\alpha} = 0 \tag{S9}$$

For a given species, the reaction terms f would therefore be a mix of positives and negatives in sign across different patches. This would partly ensure that the quantity $2f_{1,\alpha}+f_{1,\alpha+1}-2f_{1,\alpha-1}-f_{1,\alpha-2}$ is of the same order of magnitude as any of the fs. Therefore, a large D would imply that $\phi_{1,\alpha-1}-\phi_{\alpha}$ is very small so as to match the contribution from $2f_{1,\alpha}+f_{1,\alpha+1}-2f_{1,\alpha-1}-f_{1,\alpha-2}$. Note that we already have a factor of 5 with the dispersal term, which would bring down the D required to obtain sufficiently coherent densities, in contrast to the 2 patch case. A large f would just mean that higher D is needed for coherent densities. Using similar arguments, we can argue for spatially coherent densities for every species.

Since each of the f is of the form $\phi_{1,\alpha}\left(1-\phi_{1,\alpha}\right)+\phi_{1,\alpha}\sum_{j\neq 1}A_{1j,\alpha}\phi_{j,\alpha}$, therefore for coherent densities equation S9 implies the effective fixed point equation:

$$\phi_1\left(1-\phi_1\right)+\phi_1\sum_{j\neq 1}\phi_j\frac{1}{N}\sum_{\alpha}A_{1j,\alpha} \tag{S10}$$

where N is the total number of patches. This demonstrates the average interaction matrix result in the limit of spatial coherence.

S3 The case of varying carrying capacities and dispersal rates

The averaged interaction matrix provides a good approximation of the equilibrium densities, even when carrying capacities and dispersal rates are allowed to vary across species. Variance in carrying capacities can have a large effect on stability (Tang & Allesina, 2014). We test whether the averaged interaction matrix still provides a good estimate of equilibrium densities in the limit of high dispersal rates when carrying capacities are allowed to vary (Fig. S1, S2). We consider local interaction strengths with sufficiently high standard deviation, such that many species would go extinct in the absence of dispersal. If there is no spatial correlation in interaction strengths, we find that very few species go extinct as expected (Fig. S1). If the variance in carrying capacities is high, then spatial coherence is achieved at higher dispersal rates that allow for accurate predictions via the averaged interaction matrix.

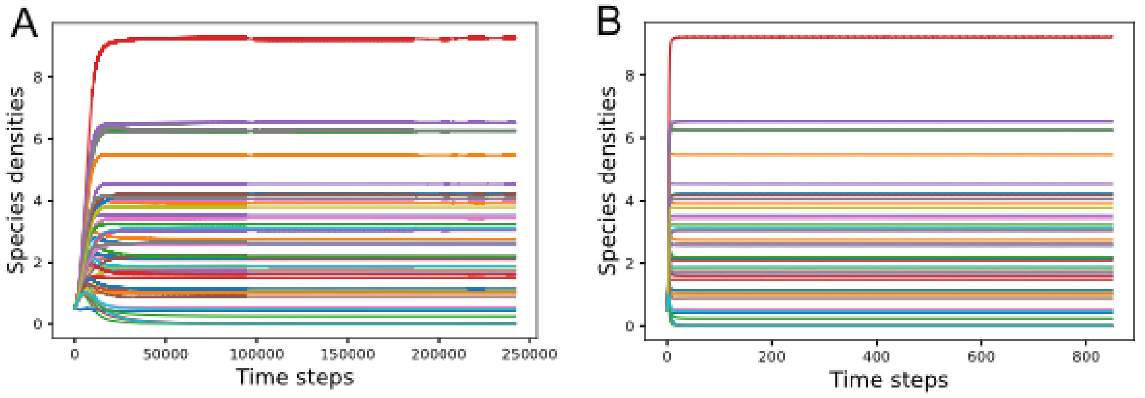

Figure S1: The case of varying carrying capacities. For fixed $D = 1000$, this plot compares species densities from the actual dynamics at patch 1 (A) versus the effective dynamics based on the averaged interaction matrix (B). The mean and standard deviation of the carrying capacities (K_i) are 3 and 1 respectively. The local interaction matrices are such that $\mu_{A_{\alpha\beta}} = 0$ and $\sigma_{A_{\alpha\beta}} = 0.1$. The number of species and the number of patches are 50 and 16 respectively.

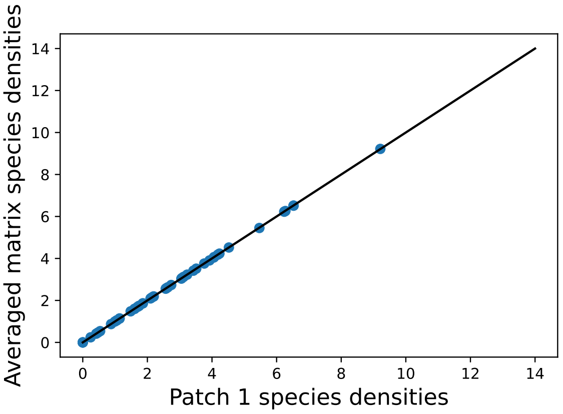

Figure S2: For the simulation settings described in Fig. S1, this plot shows the match between equilibrium species densities from patch 1 versus the corresponding estimates from the averaged interaction matrix.

We get similar results when dispersal rates are also allowed to vary across species, given that the dispersal rates are sufficiently high (Fig. S3, S4). In Fig. S1 to S4, we show comparisons with only one of the patches (patch 1), but the densities across all patches are almost the same.

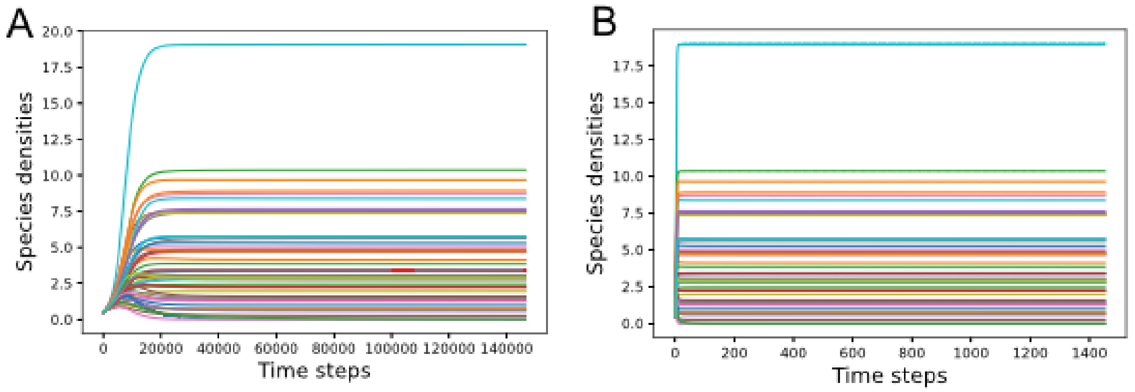

Figure S3: Varying carrying capacities as well as dispersal rates. This plot compares species densities from the actual dynamics at patch 1 (A) versus the effective dynamics based on the averaged interaction matrix (B). The mean and standard deviation of the carrying capacities (K_i) are 3 and 1 respectively. The dispersal rates are drawn from a normal distribution with mean 1000 and standard deviation 100. The local interaction matrices are such that $\mu_{A_{\alpha\beta}} = 0$ and $\sigma_{A_{\alpha\beta}} = 0.1$. The number of species and the number of patches are 50 and 16 respectively.

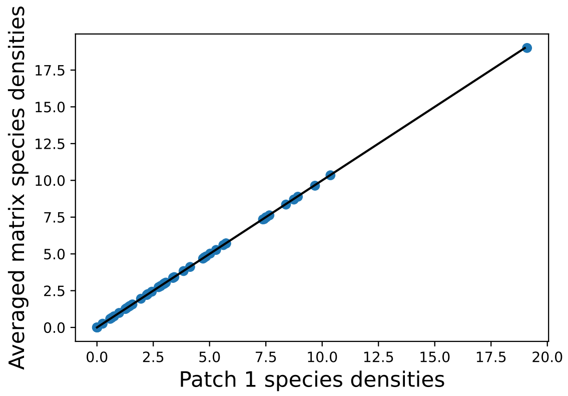

Figure S4: For the simulation settings described in Fig. S3, this plot shows the match between equilibrium species densities from patch 1 versus the corresponding estimates from the averaged interaction matrix.

S4 Derivation of the standard deviation of the effective interaction matrix

Here we derive the expressions for the standard deviation of the effective interaction matrix, $\bar{A}_{ij}$, for the cases of uncorrelated, correlated and nearest neighbour-correlated habitats respectively. The species pool for all cases is N. Starting with the uncorrelated case where each habitat has a unique random interaction matrix with standard deviation σ, mean μ and connectance (percentage of non-zero entries) c. We represent the non-zero entries in the habitat interaction matrices as stochastic variables X_{ijg}, where the indices are $i = j = 1, 2, ..N$ and $g = 1, 2...G$, with G being the number of habitats. Then the mean and standard deviation for the average interaction matrix can be found as

$$\begin{aligned} E[\bar{A}_{ij}] &= \frac{E[\sum_{g=1}^{G^*} X_{ijg}]}{G} = \frac{E[G^*]E[X_{ijg}]}{G} = \frac{cG\mu}{G} \\ &= c\mu \\ &= \bar{\mu} \end{aligned}$$

$$\begin{aligned} Var[\bar{A}_{ij}] &= \frac{Var[\sum_{g=1}^{G^*} X_{ijg}]}{G^2} \\ &= \frac{E[G^*]Var[X_{ijg}] + Var[G^*]E[X_{ijg}]^2}{G^2} \\ &= \frac{cG\sigma^2 + \mu^2 cG(1-c)}{G^2} \\ &= \frac{c(\sigma^2 + \mu^2(1-c))}{G} \\ &= \bar{\sigma}^2. \end{aligned} \tag{S11}$$

Here G^* is a binomially distributed stochastic variable for how many of the entries X_{ij} are non-zero in the habitats.

The same procedure with entries in the habitat interaction matrices as stochastic variables, X_{ijg}, is followed in the correlated and nearest neighbour-correlated case. In the correlated case however we do not need to treat the number of habitats G as a stochastic variable (G^*), since the correlation ensures that an interaction is either zero or non-zero in every habitat. For the correlated habitats we get

$$\begin{aligned} Var[\bar{A}_{ij}] &= \frac{Var\left[\sum_{\alpha=1}^{G} X_{ij\alpha}\right]}{G^2} \\ &= \frac{GVar[X_{ij\alpha}] + 2\sum_{1\leq\alpha<\beta\leq G} Cov[X_{ij\alpha}X_{ij\beta}]}{G^2} \\ &= \frac{\sigma^2\left(G + 2\rho\binom{G}{2}\right)}{G^2} \\ &= \frac{\sigma^2}{G}\left(1 + (G-1)\rho\right) \\ &= \bar{\sigma}_{\rho}^2, \end{aligned} \tag{S12}$$

Where ρ is the correlation between habitat interactions. For the nearest neighbour correlation we show the derivation for a 1-dimensional lattice. In this case there is no need for the stochastic variable G^* for the number of habitats, since all habitats are included in the sum. Instead, the strength of correlation between nearest neighbours, $\rho_{1,nn}$, next nearest neighbours, $\rho_{2,nn}$ etc. is included. The derivation for the nearest neighbour correlated habitats is shown below

$$
\begin{aligned}
Var[\bar{A}_{ij}] &= \frac{Var\left[\sum_{\alpha=1}^{G} X_{ij\alpha}\right]}{G^2} \\
&= \frac{GVar[X_{ij\alpha}] + 2\sum_{1\leq\alpha<\beta\leq G} Cov[X_{ij\alpha}X_{ij\beta}]}{G^2} \\
&= \frac{\sigma^2\left(G + 2\sum_{\eta=1}^{G-1} \rho_{\eta,nn}(G-\eta)\right)}{G^2} \\
&= \frac{\sigma^2\left(G + 2\sum_{\eta=1}^{G-1} \rho^{\eta}(G-\eta)\right)}{G^2} \\
&= \bar{\sigma}^2_{\rho_{nn}},
\end{aligned}
\tag{S13}
$$

where ρ_{nn} denotes the nearest neighbour correlation.

S5 Does topology of the spatially extended space matter?

One might suspect that the topology of the connected habitats can make a large difference in diversity of the metacommunity. To test this three types of network topologies (in addition to the grid in the main text), small world, random and Barabasi-Albert, were tested. In these cases we used a generalisation of the discrete laplace operator according to

$$\frac{\partial \phi_{i,\alpha}}{\partial t} = r_i \phi_{i,\alpha} \left(1 - \frac{\phi_{i,\alpha}}{K_i}\right) + \sigma \phi_{i,\alpha} \sum_{j=1}^{N} A_{ij,\alpha} \phi_{j,\alpha} + D_i \left(\sum_{\beta \in \{\alpha \leftarrow\}} \phi_{i,\beta} - N_{\{\alpha \rightarrow\}} \phi_{i,\alpha} \right) / h^2 \tag{S14}$$

where α is a habitat index and $\{\alpha \leftarrow\}$ denote habitats connected to α. $N_{\{\alpha \rightarrow\}}$ is the degree of habitat α (number of habitats it is connected to). The h is the distance between habitats, here set to 1.

There were crucial differences between network topologies when diffusion rates were low. We also found that a higher diffusion rate was needed to make the system spatially coherent. Although, when spatial coherence was reached no differences were found between the different network topologies and the the grid implementation.

This tells us that our representation of the effective interaction matrix is valid in the coherent limit regardless of the underlying habitat topology. On the other hand it also suggests that it might be unreasonable to assume coherence for some topologies, because of the high diffusion/dispersal rates needed. But luckily spatial coherence can be "easily" assessed, since the abundances should be approximately equal in all habitats involved.

References

Tang, S. & Allesina, S. (2014). Reactivity and stability of large ecosystems. *Frontiers in Ecology and Evolution*, 2, 21.
